# QimmeqHealth—thyroid status of Greenland sled dogs (*Canis lupus familiaris borealis*)

**DOI:** 10.1186/s13028-021-00617-8

**Published:** 2021-11-29

**Authors:** Bolette Winnerskjold Gjaldbæk, Emilie Ulrikka Andersen-Ranberg, Rikke Langebæk, Anne Kirstine Havnsøe Krogh

**Affiliations:** Department of Veterinary Clinical Sciences, Faculty of Health and Medical Sciences, University of Copenhagen, Dyrlægevej 16, 1870 Frederiksberg C, Denmark

**Keywords:** Body condition score, Management, Reference interval, Season, Sled dogs, Thyroid hormones

## Abstract

**Background:**

Greenland sled dogs (GSD) are a unique, genetically isolated population of dogs living under exceptional environmental conditions. Metabolism, and thereby thyroid hormones are affected by multiple factors. Among other activity, energy balance and environmental conditions are important. A breed-specific reference interval (RI) can be useful for diagnostics of potential thyroid-related pathologies. The aim of this study was to establish RIs of the thyroid hormones thyroxin (T4), free thyroxin (fT4), and thyroid stimulating hormone (TSH) in GSD. In addition to evaluate the effect of sex, age, season, management, and body condition score (BCS) in GSD. Physical exams and cephalic venous blood sampling were performed in the period of 2018–2019 from 265 GSD managed either privately or by the Danish navy. Serum biochemical analyses, including C-reactive protein, were performed and RIs were determined for TSH, T4 and fT4 in only healthy dogs. The RIs were determined using American Society for Veterinary Clinical Pathology guidelines and the effect of varying factors were evaluated by linear regression and further tested by Mann–Whitney test.

**Results:**

144 GSD were included in the reference group resulting in RIs: T4: 6.44–48.65 nmol/L; fT4: 3.91–18.51 pmol/L; and TSH: 0.04–0.55 ng/mL. Female GSD had significantly higher concentrations of T4 (P = 0.039) and fT4 (P = 0.015) compared to males; a positive correlation between TSH and aging was found; T4 concentrations were significantly higher (P = 0.003) during summer; and TSH concentrations were lower in GSD managed by the navy (P < 0.0001). BCS was higher (P < 0.0001) in Sirius GSD compared to civilian GSD, and BCS was positively correlated with T4 and negatively correlated with TSH.

**Conclusions:**

Reference intervals for T4, fT4 and TSH in GSD were established. The RI for T4 and fT4 was lower compared to other breeds. In addition, sex, age, season, management and BCS demonstrated variable effects on thyroid hormones. Our results can be used as a foundation for improving management and further research of GSD.

**Supplementary Information:**

The online version contains supplementary material available at 10.1186/s13028-021-00617-8.

## Background

Greenland sled dogs (GSD) is an original landrace of the Greenlandic Inuit. The Inuit of modern-day Greenland originate from an aboriginal Inuit culture that migrated to- and settled in Greenland about 900 years ago [1]. With them, they brought a relatively small number of sled dogs from which modern-day sled dogs originate. Against all odds, post colonialism, this breed has largely—and compared to other sled dog breeds outside Greenland—escaped inter-mixing with other breeds [2]. Moreover, recent studies have revealed that the GSD is the nearest living descendant to the oldest sled dog fossil recorded, discovered in Zhokov Siberia, and dated ca. 9500 years old [2]. Together, these findings describe a unique dog breed shaped by extreme demands from the environment and through their human relation, including long periods of fasting, at times a monotonous diet combined with extreme physical exertion.

The purpose of the GSD has evolved over time. Historically, the GSD were used only for subsistence hunting, and while this is still important for some GSD owners, the use now includes other activities such as racing, transportation, fishing, and tourism (Andersen-Ranberg et al. unpublished data). GSD in Greenland are however typically inactive during summer, and active during late autumn, winter, and spring, when temperatures fall, and snow covers the landscape. The dogs exhibit great stamina and the ability to transport heavy loads across long distances at extreme low temperatures and with minimal sustenance [3]. Their ability to do so, has long been to the awe of many, e.g., Arctic explorers of the nineteenth and twentieth century [4–7]. An example of this admiration—as well as indications of a unique breed’s specific function of thyroid hormones—can be found in the book “The North Pole” by Commander Robert Peary recalling his success at reaching the North Pole in 1909 [8]: “*[…] without their help [“Eskimo dogs”], success could never have crowned the efforts of the exhibition. They are sturdy, magnificent animals. There may be larger dogs than these, there may be handsomer dogs; but I doubt it. Other dogs may work as well or travel as fast and far when fully fed; but there is no dog in the World that can work so long in the lowest temperatures on practically nothing to eat.*”

This early quote indicates a dog breed that possesses a unique metabolism. Several studies have examined how metabolism is affected by prolonged endurance exercise, intense physical training as well as extreme environmental conditions [9–14]. Thyroid hormones are among the most important regulators of metabolism. These hormones are essential for maintaining energy balance and are affected by genetics, diet, environment, exercise, and ambient temperatures among other [11]. Multiple studies have reported that sled dogs generally present with lower concentrations of the thyroid hormones thyroxine (T4), free thyroxine (fT4), and thyroid-stimulating hormone (TSH) compared to other dog breeds [10–13]. It has, in this context, been suggested that low levels of thyroid hormones may be a normal breed-specific finding in these sled dog breeds, and a breed-specific reference interval would in that case be useful for precise diagnostics of potential pathologies involving thyroid-hormones [15].

This study aims: (i) to determine the concentration of thyroid hormones (T4, fT4, TSH) in healthy GSD and establish their reference intervals, (ii) to examine the behaviour of thyroid hormones in GSD under different circumstances (e.g., season, type of management, activity level), and, finally (iii) to describe the relationship between thyroid hormone concentrations and body condition score.

## Methods

### Dogs and sampling

Greenland sled dogs (> 6 months old) located in Greenland were included in the study. Information regarding age, sex, management, body condition score (BCS) [16], muscle condition score (MCS) [16], blood samples and results of a physical examination were imperative. Management for GSD were divided into two groups: GSD managed privately or by the Danish Navy. Only GSD > 6 months old were included in the study since previous data has shown a stable concentration of T4 this age [17]. Exclusion criteria for aim: (i) herein were pregnancy in the last trimester and signs of disease based on physical examination or blood biochemical analysis. Medical history and information on the present physical and behavioural status of dogs were collected from the owner.

Blood was collected by cephalic venepuncture into serum and EDTA plastic vacutainers, respectively (10 mL, BD Vacutainer). Fasting prior to blood sampling was not imperative. Analysis of T4, fT4, and TSH do not require dogs to be fasted prior to sample collection, according to the manufacturer. Blood samples were kept at 0–10 °C and was centrifuged (8000*g* in 10 min) within 4 h of collection. Whole blood, serum, and EDTA plasma were stored in cryotubes and frozen upright at − 20 °C until further analyses at Veterinary Diagnostic Laboratory, University of Copenhagen, Denmark.

Ethical approval was granted by the Departmental Ethical Administrative Committee in Greenland and by the Departmental Ethics and Administrative Committee with informed client consent.

### Sampling locations

Blood samples were collected from GSD during six separate field trips to locations in Greenland: Ilulissat, Daneborg, Qaanaaq, and Qasigiannguit. Sampling in Ilulissat and Qasigiannguit was aimed at- and timed carefully according to the annual national sled dog race, and it was thus possible to include dogs from other areas than the one visited. Field trips were carried out during summer and winter. While management and activity levels vary widely depending on owner and season, it was essential to obtain samples throughout the year and from different owners. Dogs were either owned by civilian Greenlanders or by the Danish Navy special forces, known as the Sled dog Patrol Sirius. Henceforth the GSD are referred to as civilian GSD or Sirius GSD depending on ownership. When not on route, Sirius GSD reside in Daneborg located in North-Eastern Greenland, whereas civilian GSD primarily resided on the west coast of Greenland.

### Laboratory analyses

Thyroid hormone analyses (T4, fT4, and TSH) were performed using an Immulite 2000 (Siemens Healthineers, Germany). Concentrations of T4 and fT4 were determined using a solid-phase, chemiluminescent competitive immunoassay (Siemens, Healthineers, Germany) and TSH was determined by a solid-phase, enzyme-labelled chemiluminescent immunometric assay (Siemens Healthineers, Germany). Biochemical analysis including C-reactive protein (CRP) were performed using an Advia 1800 (Siemens Healthineers, Germany). All analyses were performed following the manufacturer’s instructions. For all analytes, the analyses were performed within one analytical run.

### Statistical analyses

Statistical analyses and calculation of a 95% reference interval, including 90% confidence intervals (CI) of thyroid hormone reference limits, were carried out using freeware R 3.5.1 (R Foundation for Statistical Computing; http://stat.ethz.ch/CRAN/). Following the American Society for Veterinary Clinical Pathology (ASVCP) guidelines [18] in reference interval establishment, all reference intervals were computed non-parametrically (observed non-Gaussian distributions for T4 and fT4) [18, 19]. Distribution measurements across samples were visually evaluated by histograms, and presence of normality and log-normality tested by the D’Agostino & Pearson omnibus normality test. Suspected outliers were visually identified on depicted histograms and further evaluated by Tukey’s method.

The need for partitioning was considered for age, sex, season, type of owner (civilian GSD versus Sirius GSD), and BCS. Any difference in concentrations of thyroid hormones across these parameters was evaluated by linear regression and further tested by the Mann–Whitney test. 95% RIs and 90% CIs were computed non-parametrically.

Median values were preferred to mean values to mitigate effects of outliers as sample distributions appeared to be skewed.

## Results

A total of 306 serum samples from 265 dogs were collected during the six field trips. Out of these, 87 were excluded due to pathological findings in the physical examination and biochemical analysis. Furthermore, 57 were excluded due to lack of data (e.g., BCS, age). Among remaining samples, 18 dogs were sampled twice; to avoid dependency across observations only one sample was used, respectively. 144 dogs were deemed healthy and were included in the establishment of reference intervals for T4, fT4 and TSH (Table 1). Of the 144 dogs, 92 dogs were included from summer field trips and 52 dogs from winter field trips.

**Table 1:** Summary of sample data selection. GSD, Greenland Sled dog; Civilian, referring to dogs owned by civilian Greenlanders; Sirius, referring to dogs owned by the special Danish military unit: Sled dog Patrol Sirius

Resulting in:	Total GSD observations remaining (n)	Civilian GSD (n)	Sirius GSD (n)
1. Observations counting 265 unique GSD (Qimmeq 1–6)	306	197	109
2. 57 observations excluded due to lack of information (e.g., BCS, age)	249	42	15
3. 17 observations excluded due to findings in clinical examination (e.g., poor hair coat, dermal hyper-pigmentation)	232	17	0
4. 70 observations excluded due to findings in the biochemical analysis	162	56	14
5. 18 observations excluded to avoid duplicate GSD (winter observations kept)	144	3	15

### Reference intervals for T4, fT4, and TSH

Reference intervals were calculated for T4[6.44–48.65 nmol/L,95% lower limit CI (6.44; 6.44), 95% upper limit CI (41.7–56.00)], fT4[3.91–18.51 pmol/L, 95% lower limit CI (3.86; 4.45), 95% upper limit CI (17.1–22.70)], and TSH [0.04–0.55 ng/mL, 95% lower limit CI (0.030; 0.055), 95% upper limit CI (0.429–0.613)] (Table 2).

**Table 2:** Summary statistics and reference intervals

Serum	n	Median	Min	Max	RI (GSD)	Lower limit CI	Upper limit CI
T4 (nmol/L)	144	16.55	6.44	56.00	6.44–48.65	6.44–6.44	41.7–56.00
fT4 (pmol/L)	144	10.03	3.86	22.70	3.91–18.51	3.86–4.45	17.1–22.70
TSH (ng/mL)	143^a^	0.140	0.030	0.613	0.042–0.548	0.030–0.055	0.429–0.613

*n* number of dogs tested,* RI* Reference Interva,* GSD* Greenland Sled dog,* CI* Confidence Interval

^a^One outlier was removed for TSH

Outliers, according to Tukey’s method and visual inspection of histograms, showed one suspected outlier for T4 and fT4, respectively, and eight suspected outliers for TSH (data not shown). One outlier for TSH was excluded. According to histograms and the D’Agostino & Pearson omnibus normality test, the distribution of the reference sample group was skewed for T4 and TSH, but not for fT4.

Lower detection limits of the Immulite 2000, were 6.44 nmol/L for T4 and 3.86 pmol/L for fT4. 15 dogs (10.4% of reference sample group, nearly equally distributed between civilian GSD and Sirius GSD) presented a concentration below the analytical detection limit for T4, and three dogs (2% of the reference sample group) were below the detection limit for fT4.

### Analysis of partitioning factors

**Fig. 1:**
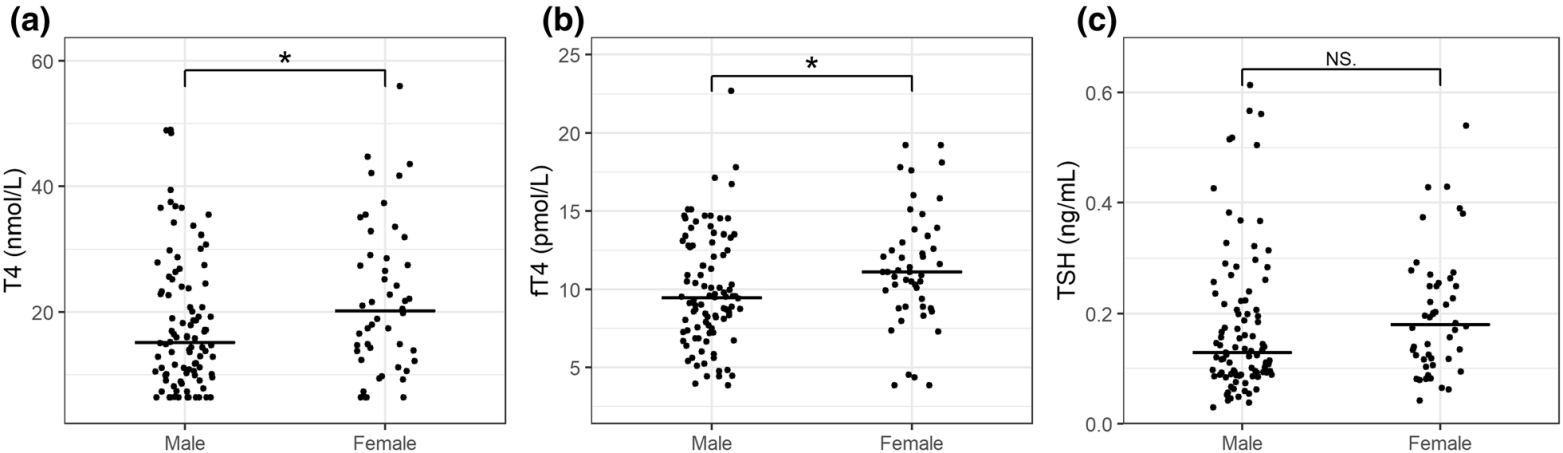
Concentrations of T4, fT4, and TSH in healthy male and female Greenland sled dogs. Horizontal lines represent medians for each group (Male/Female). Medians were significantly higher in females compared to males (P < 0.05) for T4 and fT4. Medians for TSH were not different at < 0.05 significance level. **a** T4, **b** fT4, **c** TSH

**Fig. 2:**
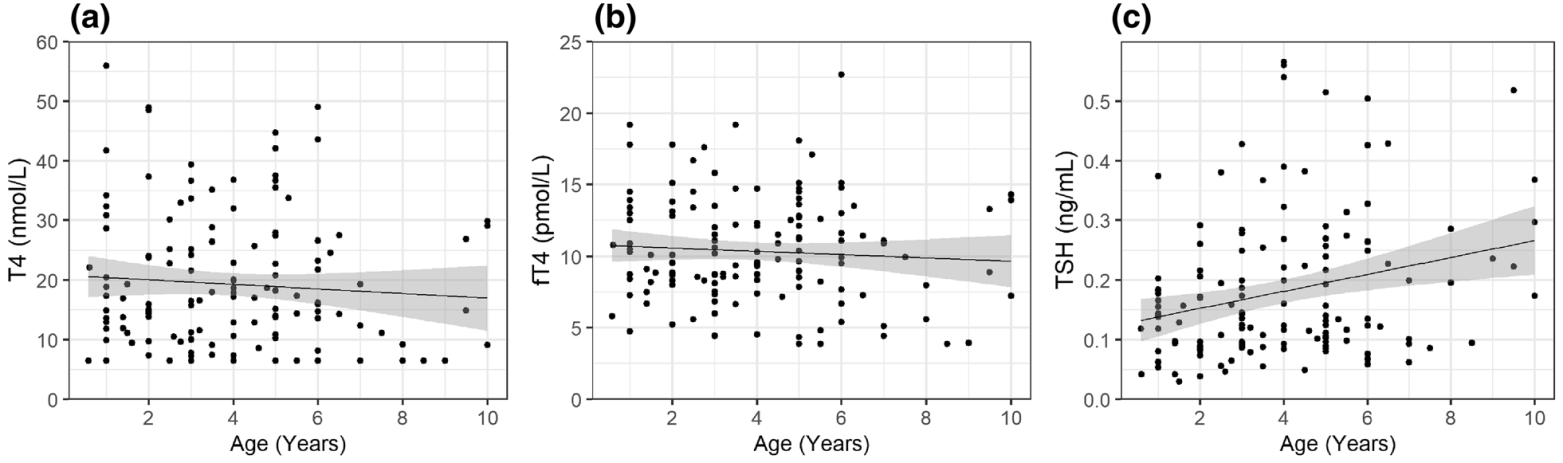
Correlation between age and T4, fT4, and TSH concentrations in healthy Greenland sled dogs. Lines represent linear correlation between age and thyroid hormones. Shaded areas denote 95% confidence intervals. A positive significant correlation with slope of 0.014 ng/mL/year is shown between age and TSH. No significant correlation was present between age and T4 and fT4. **a** T4, **b** fT4, **c** TSH

**Fig. 3:**
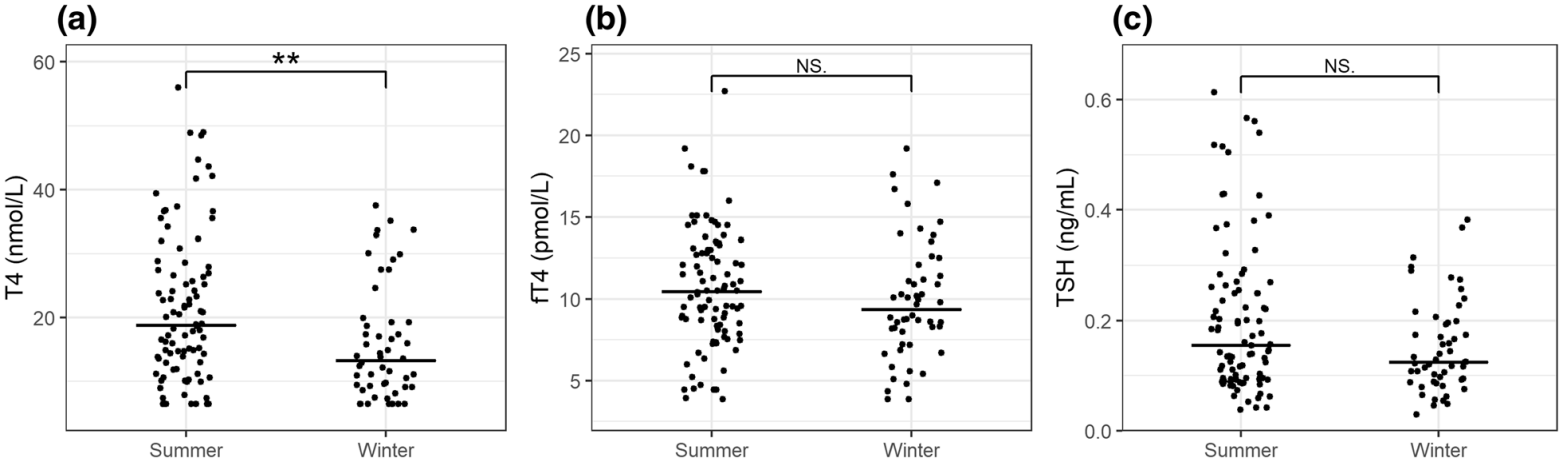
T4, fT4, and TSH concentrations in healthy Greenland sled dogs measured by season. Horizontal lines represent medians for each group. Medians were significantly higher in samples collected during summer compared to winter (P < 0.05) for T4. However, medians for fT4 and TSH were not different at a < 0.05 significance level. NS, Not significance. Significance levels: * = 0.05; ** = 0.01; *** = 0.001. **a** T4, **b** fT4, **c** TSH

**Fig. 4:**
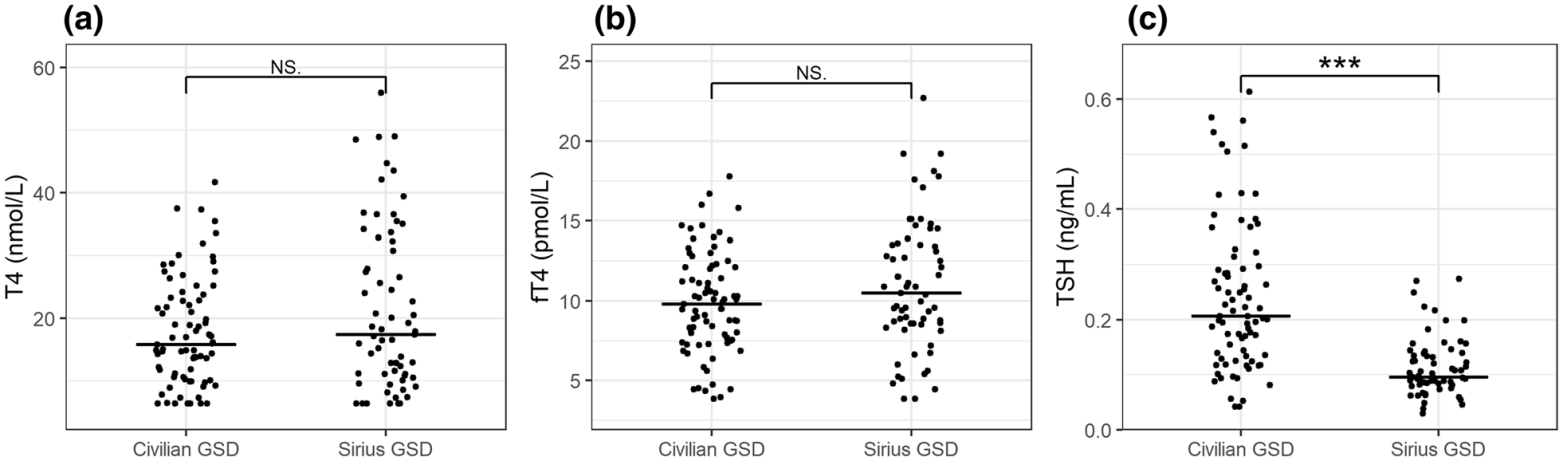
T4, fT4, and TSH concentrations in healthy Greenland sled dogs owned by Greenlandic civilians vs. the Danish Navy, i.e., Sled dog Patrol Sirius. The horizontal lines represent medians for each group. Medians were not significantly different for T4 and fT4. However, medians for TSH were significantly higher in civilian managed GSD (P < 0.05). NS, Not significance. Significance levels: * = 0.05; ** = 0.01; *** = 0.001. **a** T4, **b** fT4, **c** TSH

**Fig. 5:**
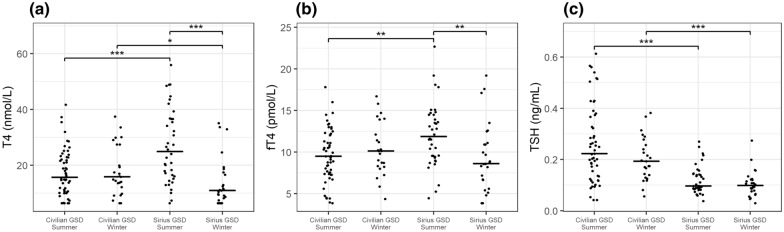
T4, fT4, and TSH concentrations in healthy Greenland sled dogs (GSD) measured by type of management (civilian GSD vs. GSD of the Danish Navy, i.e., Sled dog Patrol Sirius) and season. Horizontal lines represent medians for each group. Significant differences were observed for Sirius GSD between summer and winter for T4 and fT4, but no difference was observed for TSH. Significance levels: * = 0.05; ** = 0.01; *** = 0.001. **a** T4, **b** fT4, **c** TSH

Results are summarized in Figs. 1, 2, 3, 4, 5. Female dogs showed significantly higher concentrations of T4 (P = 0.039) and fT4 (P = 0.015) compared to males (Fig. 1). No correlation between TSH and sex was found, and likewise, age did not show effects on concentrations of T4 and fT4, but there was a significant positive correlation between concentrations of TSH and age [increasing TSH with aging, slope of 0*.*014 ng/mL/year, 95% CI (0*.*005; 0*.*023)] (Fig. 2).

Levels of T4 showed significant variation across season and was significantly higher (P = 0.003) during summer (median = 18.80 nmol/L) vs. winter (median = 13.25 nmol/L). There were no significant differences in concentrations of fT4 and TSH across season (Figure 3).

No significant differences were found for either T4 or fT4 between dogs managed and owned by civilian Greenlanders and dogs managed and owned by Sirius, but concentrations of TSH were significantly lower (P < 0.0001) in Sirius dogs (median = 0.096 ng/mL) vs. civilian (median = 0.206 ng/mL) (Fig. 4). Moreover, season affected levels of thyroid hormones differently depending on civilian vs. Sirius ownership, that is: no significant differences in T4, fT4, and TSH were observed between summer and winter for civilian GSD, but significant differences for T4 (P < 0.001) were observed for Sirius GSD between summer (median = 24.9 nmol/L) and winter (median = 10.9 nmol/L), and for fT4 (P = 0.003, summer median = 11.85, winter median = 8.61). No seasonal difference was observed for TSH in Sirius dogs (Fig. 5).

Significant differences for summer observations were found between Sirius and civilian GSD for T4 (P = 0.001, civilian GSD median = 15.65, Sirius GSD median = 24.9), fT4 (P = 0.001; civilian GSD median = 9.49, Sirius GSD median = 11.85), and TSH (P < 0.0001; civilian GSD median = 0.223, Sirius GSD median = 0.096). Furthermore, significant differences were observed for winter observations between Sirius and civilian GSD for T4 (P = 0.038; civilian GSD median = 15.8, Sirius GSD median = 10.9) and TSH (P < 0.0001; civilian GSD median = 0.193, Sirius GSD median = 0.098), but not for fT4 (Fig. 5).

### Thyroid hormones and body condition score

**Fig. 6:**
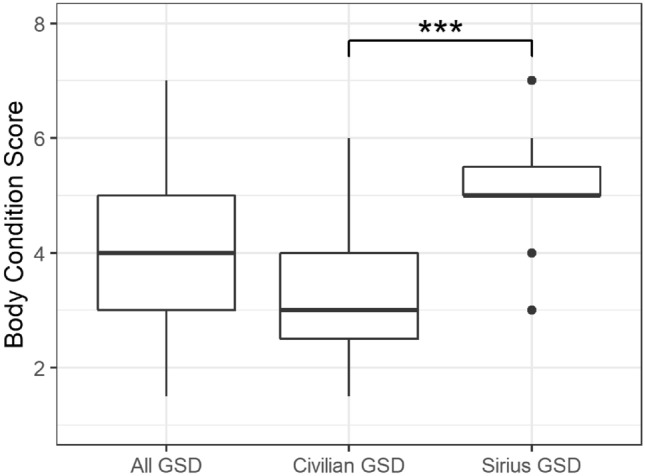
Body condition score **(**BCS) for reference individuals and by type of management. GSD, Greenland sled dogs; Civilian, referring to dogs owned by civilian Greenlanders; Sirius, referring to dogs owned by the special Danish military unit: Sled dog Patrol Sirius. Significance levels: * = 0.05; ** = 0.01; *** = 0.001

Results are summarized in Fig. 6. Median BCS for all dogs was 4/9, with no significant differences across sex and season. BCS was however significantly higher (P < 0.0001) in Sirius GSD compared to civilian GSD (Sirius GSD: median = 5 vs. civilian GSD: median = 3) (Fig. 6).

BCS was positively correlated with T4, with a slope of 1*.*46 nmol/L per BCS-point [95% CI (0*.*023; 2*.*89)], and negatively correlated with TSH, with a slope of − 0*.*043 ng/mL per BCS-point [95% CI (− 0*.*057; − 0*.*028)] (Additional file 1). There was no significant correlation between BCS and T4 or fT4 for Sirius GSD or for civilian GSD, respectively, but a positive correlation between BCS and TSH (slope of 0*.*015 nmol/L per BCS-point, P = 0.051), was observed for Sirius GSD. This finding was significant at a < 0.1 significance level, but not at a conventional < 0.05 significance level. Surprisingly, a negative correlation with a slope of − 0*.*038 nmol/L TSH per BCS-point (P = 0.03) was found among civilian GSD (Additional file 2).

### Biochemical analyses

A wide range of pathological findings were observed in the biochemical analyses. Dogs were excluded due to increased CRP, high liver enzymes [aspartate aminotransferase (AST), alkaline phosphatase (ALP), alanine aminotransferase (ALT)], high creatinine kinase (CK) and changes in electrolytes or a mix of these changes.

## Discussion

Reference intervals were established for serum concentrations of T4, fT4, and TSH in healthy GSD. Lower concentrations of T4 and fT4 were demonstrated in GSD compared to standard reference intervals based on other dog breeds. For all thyroid hormones, the width of 90% CI of lower reference limits were smaller than 20% of the width of respective reference intervals, as is recommended [18]. However, this ratio exceeded 20% for all upper reference limits. Thus, in this study, lower reference limits were estimated with a higher certainty than upper reference limits. Narrow CI of lower reference limits is likely due to the Immulite 2000’s lower detection limit, which was relatively frequently crossed, or skewness of data. Collection of additional data may contribute to a narrower CI for upper reference limits. In lack of the true value, recordings below the lower analytical detection limits were given the value of the lower detection limits. The true lower reference limit of T4 in GSD is therefore likely lower than what we have presented herein. The lower detection limit did however not affect the lower reference limit for fT4. These findings emphasize that GSD generally appear to have low concentration levels of thyroid hormones compared to other dog breeds. Low general levels of T4 and fT4 will have diagnostic consequences as the diagnosis of hypothyroidism typically relies on the ability to define a decreased level of T4 and fT4.

A previous study by Hegstad-Davies et al. [15] of seven different breeds (Alaskan Malamute, Collie, English Setter, Golden Retriever, Keeshond, Samoyed, and Siberian Husky) reported higher T4 and fT4 concentration levels compared to those reported herein for GSD [15]. The study found lowest concentration values of T4 (median = 19.3 nmol/L and fT4, median = 11.6 pmol/L) in English Setters, whereas our study indicates a median of 16.55 nmol/L for T4 and 10.03 pmol/L for fT4 in GSD. Concentration of TSH in GSD (median TSH 0.140 ng/mL) was however within the range of values observed by Hegstad-Davies et al. [15]. It is unknown why general levels of thyroid hormone fluctuate across canine breeds. Thyroid efficiency, physiological set-points, climatic stressors, and stressors related to general use, may lead to inter-breed differences, but thyroid-binding proteins and regulatory processes (e.g., thyroid hormone receptor function and affinity) may also play a role. Breed differences in other blood parameters have been reported as well [20–27]. By comparing five hunting dog breeds, Miglio et al. [20] concluded that several haematological and biochemical blood parameters were significantly different than the RI determined by previously studies for the general dog population [20]. These findings suggest that dog breeds may differ across a broader range of blood parameters increasing the demand for breed specific RIs.

In case of thyroid gland disease, it is important to be able to evaluate hormone levels against reference intervals, but a clinical diagnosis of hypothyroidism is based on *both* corresponding clinical signs and thyroid levels. Typical clinical signs of hypothyroidism may nevertheless be difficult to detect in GSD; for instance, obesity is unlikely to occur, despite pathological hypothyroidism, due to generally restricted feeding regimens. Typical clinical signs of hypothyroidism (e.g., lethargy, weight-gain, and cold intolerance) were likewise rarely observed or reported by owners among GSD in this study. Our physical examinations did detect clinical signs attributable to hypothyroidism, i.e., poor hair coat, seizures, and dermal hyperpigmentation, but these are not specific to hypothyroidism. Only a few dogs, displayed these symptoms accompanied by low T4 levels (n = 2, included seizures, hyperpigmentation, and poor coat). Altogether, evaluation of clinical signs in GSD is challenging, largely because of the type of management and the extreme environment in Greenland, and not least because of lack of veterinary assistance.

Relatively speaking, generally low thyroid levels have also been reported in other sled dog breeds [11, 13]. This does not make them immune to hypothyroidism, however, as is evidenced by the finding of typical and debilitating clinical signs of hypothyroidism in sled dog (mix) breeds accompanied by low T4 levels. Some of these sled dogs already display these clinical signs at T4 levels around 10–13 mmol/L (pers. comm. Celine Laforet, DVM and sled dog race veterinarian, Tromsø, Norway). This suggests a potential for pathologically low thyroid levels in GSD too, which may be masked by the aforementioned challenges of identifying clinical signs and lack of veterinary assistance. There is thereby also the possibility that hypothyroid dogs have been included in our reference group. We therefore recommend that occurrence of pathological hypothyroidism in GSD should be studied further. Nevertheless, correspondingly low thyroid hormone levels in other healthy sled dog breeds [11, 13], suggest that pituitary and thyroid set-points may generally differ for Arctic sled dog breeds, compared to other dog breeds.

The need for partitioning of dogs into distinct groups was assessed according to the ASVCP reference interval guidelines [18], i.e. partitioning is advised when a significant difference of group medians is larger than 25% of the combined reference interval. Results showed a significant difference for sex (T4, fT4), age (TSH), season (T4), management (TSH), and BCS (T4, TSH), however, none of the differences of the medians exceeded 25% of the range of the combined reference interval, so no further partitioning in establishing a reference interval for this breed was considered.

The sex-associated variation in T4 and fT4 concentrations is consistent with a previous study including a variety of breeds [15]. Furthermore, increased T4 concentration has been reported in dioestrus and pregnant bitches, compared to bitches in other reproductive states, and males [28]. Therefore, concerning each individual dog included knowledge on reproductive state would have been ideal. Diversely, there were no sex-associated variations in TSH, which is also in accordance with similar studies [15].

A positive correlation between age and concentrations of TSH, T4 and fT4 have previously been reported in dogs [15], in our study it was only found for TSH. GSD were considered adult from the age of six months when the dogs start to work. Previous data support this choice of age limit since a stable concentration level of T4 have been demonstrated from the age of six months in dogs [17]. In addition, our analyses did not reveal significant variations of T4, fT4 and TSH in dogs younger than one year, compared to older dogs, indicating that the youngest dogs included in our study did not bias our results.

Median BCS was 3/9 and 5/9 for civilian and Sirius GSD, respectively. This difference is most likely due to different feeding regimens, de-worming regimens and not least diverging views on optimal BCS for GSD (Andersen-Ranberg et al. unpublished data). Furthermore, Sirius GSD have access to veterinary consultancy and care, unlike the majority of civilian GSD. BCS was obtained by different people throughout the study but general protocols were followed to minimize potential biases [16].

Overall, a positive correlation was found between BCS and T4, and a negative correlation was found between BCS and TSH. No such relationship has been identified in the literature before, and it is argued that the results should be interpreted with care as there may be other factors affecting BCS and thyroid hormone levels simultaneously. Importantly, type of management was shown to affect levels of TSH considerably, i.e., focusing on Sirius GSD, an increasing trend (P < 0.1) for TSH with increasing BCS was demonstrated, which is opposite that of the overall significant result. A significantly higher BCS in Sirius GSD compared to civilian GSD possibly contributes to this discrepancy, but this assumption cannot be confirmed from our available data alone. Further work examining why civilian GSD differ from Sirius GSD regarding conflicting correlations between BCS and TSH is prudent and may help us understand specific coping mechanisms of these dogs, as well as the role and behaviour of thyroid hormones in general.

Overall, this study showed significantly higher concentration levels of T4 in samples collected during summer compared to winter samples. Previous studies have observed fluctuations in thyroid hormone concentration levels throughout the year for other breeds [29, 30]. No studies have however examined the concentrations of thyroid hormones in dogs living in similar environmental conditions to the GSD.

Estimating effect of season is challenging for GSD in Greenland as their activity level is also highly dependent on season. GSD are largely confined to a chain during summer and work (and otherwise chained) during winter. Comparisons of activity levels during winter were difficult due to variable types and intensity of activities among civilian dogs (e.g., race dogs vs. dogs used for leisure). Activity level of Sirius GSD is generally very homogenous. They are used for long-range patrols up to 4500 km, pulling 300–550 kg, over 6 months (typically *en route* 6 h or 30 km per day). This makes it difficult to partition groups of dogs into different activity levels and the strong connection between season and activity level makes it challenging to conclude whether the estimated correlation is due to variation in season (e.g., temperatures), activity levels, or a combination of both. Optimally, effect of activity level should be measured for samples obtained in the same season.

Other relevant factors to consider include temperature, hours of daylight and diet. Studies have shown a decrease in thyroid hormone concentrations when humans are exposed to cold [31, 32]. GSD are however bred, raised, conditioned, and trained in low temperatures, suggesting that lower concentration of T4 during winter could be attributed to activity, and not to necessarily the exposure of a cold climate. Correspondingly, other studies have shown an association between exercise and a significant decrease in concentration of thyroid hormones [10, 12, 13, 33]. These findings suggest that the reduction of T4 and fT4 concentration levels observed for Sirius GSD during winter may be due to changes in activity level. It remains unclear why civilian GSD are not affected similarly; a discrepancy in activity level during winter may be at least partly accountable, but the question is nevertheless worth pursuing in future research.

Management of GSD differs significantly from most other dog breeds. GSD managed by civilian Greenlanders are typically chained year-round when not pulling sleds. The activity level of civilian GSD varies widely. Some civilian dogs are rarely activated at all and spend almost their entire life chained. Some dogs are slowly trained for pulling the sled, every autumn, and some dogs experience a sudden change in activity as they are used for pulling sleds immediately after the first snow has fallen. Sirius GSD typically maintain a more stable activity level throughout the year, although their activity levels also decrease during summer, compared to the activity extremes of winter and spring. This study found no correlation between management and T4 and fT4 but observed a strongly significant correlation between type of management and TSH, with significantly higher TSH concentration levels in civilian GSD. This is likely due to feeding differences and/or access to veterinary care.

In terms of diet, a high concentration of iodine in marine animals has been documented as seal and whale [34]. High intake of iodine can impair thyroid hormone concentration and decrease the concentration of T4 and fT4 known as the Wolff-Chaikoff effect [35]. The high concentration of TSH in civilian GSD may be affected by a high intake of iodine from a diet consisting of marine animals, inhibiting the synthesis of thyroid hormones causing low levels of T4 and fT4. When T4 and fT4 decrease, TSH correspondingly increases to stimulate the production of thyroid hormones [35]. Feeding marine mammal meat is however decreasing and the use of pelleted dry feed increasing among civilian GSD. A more detailed analysis of diet and thyroid hormones is needed.

### Some limitations should be considered for this study

Health evaluation could have been supported by tests such as complete blood count, urinalysis, and faecal sample analyses. These were largely excluded for practical reasons. Furthermore, a thyroglobulin autoantibody (TgAA) test was not conducted due to financial limitations. Elevated TgAA concentrations are rare (< 1%) but occur. TgAA artificially elevates T4 measurements even when the dog may in fact be hypothyroid [36]. Moreover, a study suggests that TgAA is breed-specific as well [15]. When comparing summer/winter it is ideal to use repeated samples. Data for both seasons, regarding the same dog, was however only available for 18 GSD, and using different dogs to compare seasonal variation and trends may impose a bias. Another limitation is that potential seasonal effects can be caused by a broad range of variables e.g., temperature, daylight, activities, feeding, which are difficult to distinguish.

## Conclusions

Reference intervals for the thyroid hormones T4, fT4 and TSH have been established and the effect of sex, age, season, management, and BCS have been assessed. These new data can be used for evaluating thyroid hormone profiles from e.g., clinically diseased GSD in the future. Throughout this study, results showed a difference between civilian and Sirius GSD but separate RI were not established based on guideline recommendations [18], moreover, these dogs are not genetically diverse, but merely managed differently. Future research concerning health and thyroid status of GSD should carefully consider the type of management.

Our results can inform veterinarians, caregivers, and owners on improved management of GSD, including assessment of thyroid-related diseases and/or conditions. Further studies are however needed focusing on susceptibility or potential resistance, including potential breed-specific clinical signs, of GSD to thyroid-related disease, hypothyroidism in particular. Overall, our results also shed light upon the function and behaviour of thyroid hormones in a mammalian species, in an extreme environment, and under multiple stressors.

## Supplementary Information

**Additional file 1.** Correlation between body condition score (BCS) and T4, fT4, and TSH concentrations in healthy Greenland sled dogs. Lines represent linear correlation between age and thyroid hormones. Shaded areas denote 95% confidence intervals. T4 and TSH were found to be correlated with BCS with slopes of 1.46 nmol/L per BCS-point and − 0.043 ng/mL per BCS-point, respectively. There was no correlation between BCS and fT4. a) T4, b) fT4, c) TSH.

**Additional file 2.** Correlation between body condition score (BCS) and thyroid stimulating hormone (TSH) concentrations in healthy Greenland sled dogs measured by type of management (Sirius (a), civilian (b)). Lines represent linear correlation between age and thyroid hormones. Shaded areas denote 95% confidence intervals. Civilian, referring to dogs owned by civilian Greenlanders; Sirius, referring to dogs owned by the special Danish military unit: Sled dog Patrol Sirius. TSH was found to be correlated with BCS with slopes of 0.015 nmol/L per BCS-point and − 0.038 ng/mL per BCS-point for Sirius GSD and civilian GSD, respectively.

## Data Availability

The datasets used and analysed during the current study are available from the corresponding author on reasonable request.

## Abbreviations

ALP: Alkaline phosphate
ALT: Alanine transaminase
AST: Aspartate aminotransferase
BCS: Body condition score
CI: Confidence interval
CK: Creatine kinase
CRP: C-reactive protein
fT4: Free thyroxine
GSD: Greenland sled dog
MCS: Muscle condition score
RI: Reference interval
T4: Thyroxine
TgAA: Thyroglobulin autoantibodies
TSH: Thyroid stimulating hormone
