# Supplementary figures and images for: QimmeqHealth—thyroid status of Greenland sled dogs (Canis lupus familiaris borealis)
Source: Acta Vet Scand. 2021 Nov 29;63:51. doi: 10.1186/s13028-021-00617-8 (PMC8630915; doi:10.1186/s13028-021-00617-8)

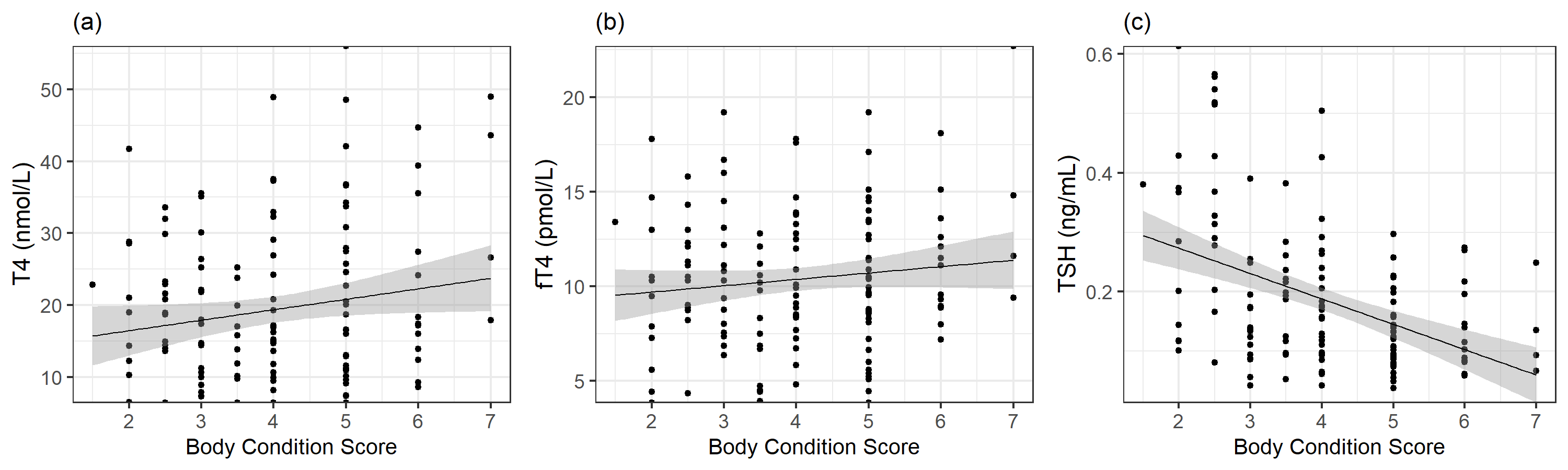

Supplement: Supplementary file 1 — Additional file 1. Correlation between body condition score (BCS) and T4, fT4, and TSH concentrations in healthy Greenland sled dogs. Lines represent linear correlation between age and thyroid hormones. Shaded areas denote 95% confidence intervals. T4 and TSH were found to be correlated with BCS with slopes of 1.46 nmol/L per BCS-point and − 0.043 ng/mL per BCS-point, respectively. There was no correlation between BCS and fT4. a) T4, b) fT4, c) TSH. [file 13028_2021_617_MOESM1_ESM.png]

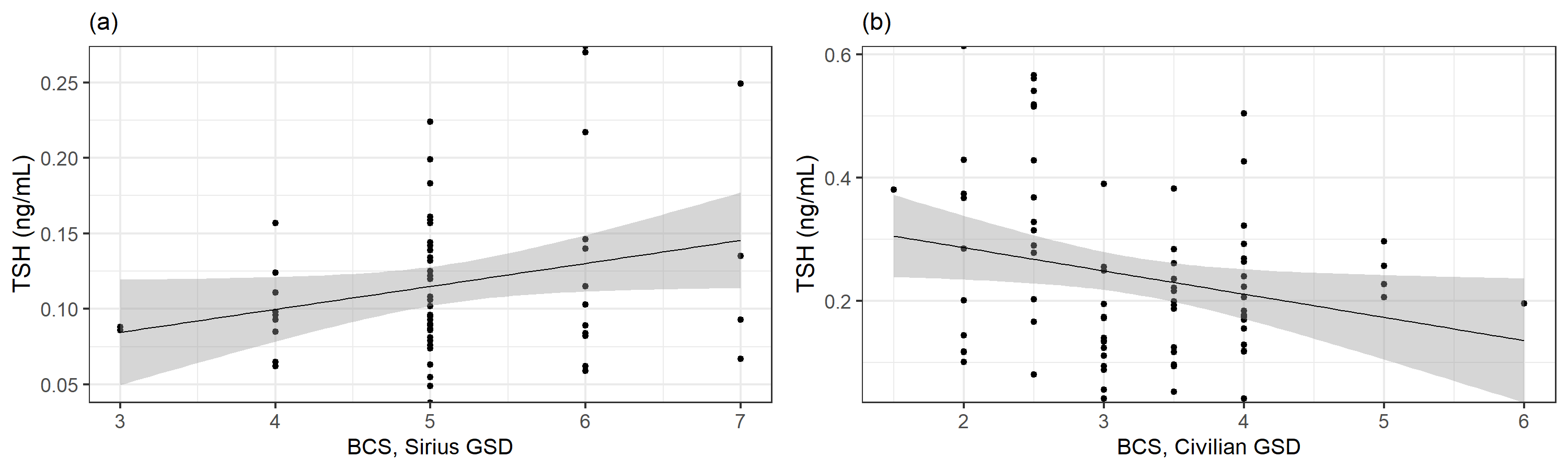

Supplement: Supplementary file 2 — Additional file 2. Correlation between body condition score (BCS) and thyroid stimulating hormone (TSH) concentrations in healthy Greenland sled dogs measured by type of management (Sirius (a), civilian (b)). Lines represent linear correlation between age and thyroid hormones. Shaded areas denote 95% confidence intervals. Civilian, referring to dogs owned by civilian Greenlanders; Sirius, referring to dogs owned by the special Danish military unit: Sled dog Patrol Sirius. TSH was found to be correlated with BCS with slopes of 0.015 nmol/L per BCS-point and − 0.038 ng/mL per BCS-point for Sirius GSD and civilian GSD, respectively. [file 13028_2021_617_MOESM2_ESM.png]
